# Adipose tissue gene expression and longitudinal clinical phenotypes are early biomarkers of lipid-regulating drug usage

**DOI:** 10.1038/s41598-025-13693-x

**Published:** 2025-08-29

**Authors:** Max Tomlinson, Julia S. El-Sayed Moustafa, Xinyuan Zhang, Yasrab Raza, Dongmeng Wang, Alan Hodgkinson, Kerrin S. Small

**Affiliations:** 1 Department of Medical & Molecular Genetics, School of Basic and Medical Biosciences, King’s College London, London, UK; 2 Department of Twin Research & Genetic Epidemiology, King’s College London, London, UK

**Keywords:** Adipose tissue, Cardiovascular disease, Lipid dysregulation, Lipid metabolism, Transcriptomics, Gene expression, Transcriptomics

## Abstract

Cardiovascular disease progression is characterised by the dysregulation of lipid metabolism and pro-atherogenic effects of adipose tissue signalling. Recent findings from the analysis of transcriptomic data in bulk tissue has enabled these insights and revealed important changes in gene expression. However, few studies have explored these molecular mechanisms before the onset of cardiovascular disease. We explore associations between future lipid-regulating drug use and cardiometabolic traits (*n* = 103), including DXA scans of body composition at baseline and follow-up 5–10 years later, in a cohort of British twins (*n* up to 6963). Utilising transcriptomic profiles from a subset of twins (*n* = 766), we explore the associations between baseline adipose tissue gene expression, clinical traits, and future lipid-regulating drug usage. We then test the joint predictive capacity of clinical traits plus gene expression compared to traditional risk scores using an automated machine learning approach. We find 44 traits are associated with lipid-regulating drug usage including measurements of abdominal fat tissue, cardiovascular health, and lipid metabolism (FDR 5%). Then, we present that adipose tissue gene expression levels at baseline are associated cross-sectionally with 19 of these 44 traits (FDR 5%). By comparing adipose gene expression levels between individuals prescribed lipid-regulating drugs in the future and controls, we discover that genes associated with 16 of these 19 traits produced greater log_2_-fold changes, suggesting shared mechanisms. We reveal 15 differentially expressed genes comparing future lipid-regulating drug users and controls at baseline (FDR 10%), including some implicated in angiogenesis: *ESM1*, *RCAN2*, and *SOCS3*. Functional enrichment with 1212 significantly differentially expressed genes (*p* < 0.05) included molecular mechanisms related to abnormal cardiovascular system electrophysiology (*p* = 1.89 × 10^−3^), arrhythmia (*p* = 4.02 × 10^−3^), and mitochondrial pathways (*p* = 1.12 × 10^−3^). Finally, we confirm inclusion of gene expression levels as features in machine learning models achieves a better AUC (0.919) compared to traditional risk predictors. These findings highlight the potential of bulk transcriptomic data to improve risk stratification for lipid-regulating drug use, offering new insights into the RNA biology of adipose tissue and advancing approaches for cardiovascular disease prevention.

**Supplementary Information:**

The online version contains supplementary material available at 10.1038/s41598-025-13693-x.

## Introduction

Cardiovascular disease (CVD) is the leading cause of mortality worldwide and accounted for approximately 32% of deaths in 2019, despite estimations that up to 90% of cases might be preventable^1^. CVD is a term for conditions that influence the heart and the blood vessels, which are characterised by progressive build-up of atherosclerotic plaques in arteries^2^. Recent evidence supports this emerging picture that CVD is driven by interactions between genetic and environmental factors, which influence the accumulation of unrepaired damage^3^. Epidemiological studies of coronary artery disease (CAD) have produced estimates of genetic heritability between 40 and 50%, while GWAS have located over 300 genetic loci that explain 30–40% of the heritability and have been implicated in angiogenesis, inflammation, and lipid metabolism^4^. Decades of study has shown that age, sex, hypertension, diabetes, obesity, and dysregulated lipid metabolism increase risk of CVD^5^. These risk factors are used clinically to identify people that may benefit from lipid-regulating drugs, but detailed longitudinal characterisation has been consistently lacking.

Obesity is a major risk factor for CVD and affects more than a quarter of adults in the UK^6^. Excess fat exacerbates ‘hallmarks’ of ageing including genomic instability, telomere erosion, epigenetic alterations, loss of proteostasis, mitochondrial dysfunction, cellular senescence, and stem cell exhaustion, which triggers immune responses that promote the accumulation of atherosclerotic plaques^7^. Longitudinal studies have showed strong evidence of changes in these hallmarks for individuals with atherosclerotic plaques, including increased levels of inflammatory proteins such as C-reactive protein and Interleukins. Importantly, these have been shown to be predictive of CVD risk^8^, suggesting obesity and ageing hallmarks could hold potential for improving clinical health outcomes^9^.

Adipose tissue is known as an endocrine organ and immune system regulator for its role in producing hormones and proinflammatory cytokines^10^. The distribution of adipose tissue in both the subcutaneous and visceral depots, plus in both the android and gynoid regions, correlates with the progression of pathologies associated with CVD^11^. Metabolic changes influenced by increased adiposity can have profound effects on dyslipidaemia (higher levels of triglycerides and low-density lipoprotein, alongside low levels of high-density lipoprotein), as engorged adipocytes become saturated then rupture, circulating atherogenic adipokines and lipids into the blood^12^. Several indicators suggesting pathological changes have been found such as apoptosis, inflammation, LDL production, and oxidative stress markers^13^, while RNA-sequencing studies have elucidated markers connected with CVD pathology^14^. Yet, there is a growing need for studies that profile the underlying transcriptional processes happening *prior* to the development of lipid dysregulation and CVD, especially as detection of biomarkers could help prevent disease in high-risk individuals^15^.

In this study, we explore the associations between clinical traits related to CVD progression, molecular processes in adipose tissue, and future prescription of lipid-regulating drugs using longitudinal clinical phenotyping and transcriptomic data from TwinsUK. We find differences between many cardiometabolic and obesity traits and adipose tissue gene expression levels several years prior to individuals being prescribed lipid-regulating drugs and determine that combining these features discriminates outcomes better than traditional markers.

## Methods

### Study design

In this study, we planned to characterise the phenotypic and molecular changes happening prior to lipid dysregulation. We used participants from TwinsUK, the largest cohort of adult twins in the UK. The registry comprises over 16,000 twins, the majority of which are female (82%) and middle-aged (median age 60). Over the last thirty years, TwinsUK has collected detailed questionnaire responses from routine clinical visits to the Department of Twin Research and Genetic Epidemiology, which is located at King’s College London^16^.

Participants self-report usage of prescription medication in questionnaires at least every 3–4 years, which allows free‐text drug entries as well as selection from a drop-down list of drug classes. These inputs were matched with British National Formulary (BNF v01-01-2019) records and reviewed in-house by a clinician^17^. As a result, the lipid-regulating drug class would have medical substances including (but not exclusively): Bezafibrate, Cholestyramine, Ezetimibe, Statins, and other lipid-regulating preparations (ATC C10A/C10B).

Our study design included 8195 participants which had responded to multiple questionnaires and attended clinical visits between 2001 and 2019. Participants were placed into 2 groups: people on prescribed lipid-regulating drugs (cases) or not (controls). Participants prescribed drugs between 2001 and 2010 were defined as having lipid dysregulation at ‘baseline’ and those on drugs between 2014 and 2019 (but not ‘baseline’) were defined as having lipid dysregulation during ‘follow-up’ (Supplementary Table 1). As we did not have access to the precise date of first prescription of lipid-lowering medication, thus lipid-regulating drug use was inferred from the next available questionnaire response from 2014 or later.

Prescriptions were used as a proxy for lipid dysregulation as they should involve evaluations by a medical professional and are usually prescribed after routine blood tests that quantify circulating lipids^18^. These classifications enable cross-sectional analysis to compare users at baseline with controls, and longitudinal analysis for comparison of participants that were subsequently prescribed lipid-regulating drugs during follow-up versus controls.

### Study materials

TwinsUK collects comprehensive longitudinal measurements of many clinical, biochemical, behavioural, dietary, and sociological factors, providing scientists with data for the analysis of complex age-related traits. Measurements are taken at routine clinical visits to TwinsUK, allowing us to explore statistical associations between drug-prescription classifications and clinical traits measured concurrently. A list of 180 clinical traits was compiled from TwinsUK incorporating blood pressure, body fat distributions from dual-energy X-ray absorptiometry, glucose and insulin, lipid profiles, height, and weight. Diabetes status was determined with questionnaire data and fasting blood glucose and insulin levels. Participants were classified as diabetic if they either reported taking diabetes medication, or their fasting blood glucose was greater than 7 mmol/L during at least one of their clinical visits, or self-reported having been diagnosed with diabetes by a medical professional. Lipids including total cholesterol, HDL, LDL, and triglyceride levels were then calculated with a colorimetric enzymatic method (mmol/L)^19^. In addition, HDL was estimated by precipitating large lipids with magnesium and dextran sulphate, and LDL was calculated with Friedewald’s equation. The frailty index was calculated as the proportion of the age-related health deficits reported by participants using over 36 general health domains such as chronic health conditions, physical inactivity, cognitive function, and social isolation^20^. Age in years, total cholesterol, HDL cholesterol, blood pressure, hypertension drug use, smoker status, and diabetes were used to calculate atherosclerotic cardiovascular disease (ASCVD) risk^21^ (Supplementary Table 2).

### Data pre-processing

Strongly correlated DXA adiposity measurements (*r* > 0.9) and those with near zero variance were filtered first with the ‘findCorrelation’ function from the caret package (version 6.0–92). For glucose and insulin, participants that had not fasted prior to clinical visit were removed. Then, clinical traits were filtered by year to obtain their first result between 2001 and 2010, leaving 103 in total, then outliers were removed using the formula below:

$$\:I=[\:q0.25-1.5\cdot\:IQR;q0.75+1.5\cdot\:IQR\:]$$

Next, for representing the trajectories of clinical traits between visits, we extracted residuals from linear models, which fitted the raw differences in the first (between 2001 and 2010) and last (between 2014 and 2019) recorded values versus time difference between visits, and controlling for baseline as this is correlated with value at follow-up^22^:

$$\:Raw\:difference\:\sim\:time\:difference\:in\:days+result\:at\:baseline$$

Transcriptomic profiles were available for 766 participants with samples from adipose tissue, with between 152 and 760 matching baseline clinical trait results (median age: 60, range: 38–64; median BMI: 25, range: 16–47). The RNA-sequencing data production and pre-processing are described in the reference here^23^. Succinctly, sub-umbilical subcutaneous adipose tissue punch biopsies were taken from participants between 2007 and 2009. STAR software (v2.4.0.1) was used for aligning properly paired short reads to the ‘hg19’ reference genome. Reads with mapping quality less than 10 were filtered out^24^. Gene expression was quantified by using ‘featureCounts’ with the GENCODE annotation (v19) and trimmed mean of M-values-adjusted^25^. Finally, expression was filtered by 5 counts or more in at least 25% of samples to exclude genes with lower expression while reducing the impact of outliers, inverse-rank normalised within each gene to stabilise variance, and the genes failing a Shapiro-Wilk test of normality (Bonferroni-adjusted p-value) were removed to avoid violating linear model assumptions.

### Phenotype analysis

All statistical analysis was performed in R (version 4.2.2) and high-throughput analyses were processed on the research computing infrastructure at King’s College London, UK (CREATE). For 103 clinical traits at baseline, data were available for between 108 and 3,510 participants. (Supplementary Table 3). Residuals taken from linear models of 88 clinical trait trajectories for those with multiple measurements were included in the list of traits. Next, we carried out a case-control study to investigate the association between future lipid-regulating drug use and various clinical traits, adjusting for age and BMI at baseline, the year of visit at baseline, year of birth, sex, zygosity, and family ID as a random effect. The analysis was implemented by using the ‘glmer’ function from the lme4 package (version 1.1–29):

$$\:Future\:medication\:use\:\sim\:clinical\:trait+age+BMI+$$

$$visit\:year+year\:of\:birth+sex+zygosity+\left(1\right|family)$$

The ‘optimx’ optimiser version (2022 − 4.30) with the nlminb minimiser was used for handling complex mixed models with random effect parameters. Models were tested in parallel using ‘mclapply’ from parallel (version 3.4.0) and compared with models where clinical traits were omitted. P-value adjustment with was applied using qvalue (version 2.22.0)^26,27^.

### Gene expression association study

To identify molecular processes in adipose tissue associated with lipid-regulating drug usage, a gene expression association study was run for normalised gene expression values against clinical traits associated with future lipid-regulating drug use. For the clinical traits reflecting lipid levels (e.g., cholesterol), participants which reported baseline lipid-regulating drug use were removed prior to analysis to avoid capturing treatment effects. Linear mixed models of TMM-normalised gene expression values against clinical traits were used to accommodate continuous outcomes and model random effects such as family structure. RNA-sequencing covariate selection (age, BMI, insert size median, GC mean, primer index, sequencing data) was based on previous work identifying the technical factors that best captured common axes of variation in the gene expression data^28^:

$$\:Gene\:expression\:\sim\:clinical\:trait+age+BMI+insert\:size\:median+GC\:mean+\left(1\right|primer)+$$

$$(1\left|date\right)+\left(1\right|zygosity)+(1\left|family\right)+\left(1\right|batch)$$

Significant associations were identified by comparing each model with the null model using ANOVA where clinical traits were omitted. Models were compared to another model where clinical traits were omitted with an ANOVA, and p-values were extracted, indicating if these clinical traits were improving the model fit and corrected with qvalue (FDR threshold at 5%).

### Pathway analysis

P-values of association between adipose tissue gene expression values and clinical traits or future lipid-regulating drug usage were filtered using the Benjamini-Hochberg procedure or testing procedure called Independent Hypothesis Weighting that optimises for power^29^. Gene names were retrieved using biomaRt (version 2.54.1), while Ensembl IDs were entered directly into gprofiler2 for enrichment analysis using a background of all the genes tested in adipose tissue (version 0.2.1). Finally, p-values of functional enrichment were corrected with the in-built ‘gSCS’ correction method, then associated terms and processes were aggregated across clinical traits to identify the molecular markers that were most strongly enriched.

### Differential expression analysis

DESeq2 was used to assess significant differences in gene expression between future users of lipid-regulating drugs (cases) and controls (DESeq2 version 1.30.1)^30^. As DESeq2 cannot currently support the use of random effects, family effects were controlled for by censoring one twin per family and preferentially selecting cases (Supplementary Table 4). Genes were prefiltered for 10 or more counts in the minimum group sample size, normalised by DESeq2’s median-of-ratios sizing factor and dispersion estimation, then transformed using an in-built variance-stabilising method (rlog)^30^. Sample outliers were removed using approximation of projection pursuit estimators calculated with ‘PcaGrid’^31^ (rrcov version 1.6-0). Diabetes status was also included as previous work in the lab showed this can alter gene expression, potentially confounding the effect of future medication usage. For this analysis, the adjusted p-value threshold was set to 0.1 (FDR 10%) to prioritise detection of true positives (sensitivity) from the weaker biological signals anticipated when detecting differences in gene expression years before medication use. Diabetes status is included due to its confounding influence on expression, but this does not account for any heterogeneity in subtypes or varied progression.

$$\:Gene\:expression\:\sim\:age+BMI+diabetes+future\:medication\:use$$

In parallel to the DESeq2 analysis, differential expression analysis was also performed with limma-voom^32^. These raw counts were pre-filtered as before, and TMM-normalised with calcNormFactors and converted into log_2_-counts-per-million (log_2_-CPM). Precision weights were computed by modelling the mean-variance trend in the data and applied to transform the values to make them suitable for linear modelling^33^. Empirical Bayes moderation was applied to shrink estimates. To assess concordance between these methods, the genes with nominal p-values were compared (*p* < 0.05), calculating the percentage of concordant genes and squared Pearson correlation (*R*) between log-fold changes. Moreover, in 510 TwinsUK participants with both adipose tissue RNA-seq and DNA methylation data, the deviation of Horvath epigenetic age from chronological age was computed after controlling for age^34^, details of which are described in this previous publication^19^.

Differential expression analysis results were combined by gene ID with results obtained from the association analyses between gene expression and clinical traits. Gene expression values associated with clinical traits that passed an FDR 5% were categorised as either ‘significant’ (q < 0.05) or ‘not significant’ (q > 0.05). To validate specific genes in adipose tissue that were differentially expressed and linked with clinical traits preceding future lipid-regulating users, we compared the absolute log_2_-fold change from the differential expression analysis between ‘significant’ and ‘not significant’ genes identified from the association analysis, determined using a Wilcoxon test.

### Data pre-processing for machine learning

To assess the predictive power of baseline clinical traits and adipose tissue gene expression for future lipid-regulating drug usage, we applied various Machine Learning algorithms using our results and previous research to guide feature selection^35^. The models were selected automatically from a list of generalised linear models, XGBoost (Extreme Gradient Boosting), GBM (Gradient Boosting Machines), as well as stacked ensembles of these models with random grid search over the hyperparameters specific for each variety of model. We used different feature combinations: (1) the ASCVD risk scores, (2) 28 baseline clinical traits which were significantly associated with future lipid-regulating drug usage, gene expression values from the genes differentially expressed between future cases and controls at two thresholds: (3) FDR 5% (*n* = 15) and (4) p-value 0.05 (*n* = 1212), and (5) all features together.

### Automated machine learning with H2O

We used an automated machine learning workflow to test these machine learning models, which is fully described here: https://docs.h2o.ai/h2o/latest-stable/h2o-docs/automl.html. The pre-processed dataset was split into training, cross-validation, and test sets with a ratio of 70–15–15 (*n* = 251–39–54) by utilising the ‘h2o.splitFrame’ function (H2O version 3.44.0.2). In this AutoML pipeline, we defined the predictor variables (*x*), identified the target variable (*y*), designated the training, cross-validation, and training datasets, then, most importantly, the maximum number of models to be trained (*n* = 100), utilising a 10-fold cross-validation, and for replicability and model interpretability, deep neural networks were discounted from the available models. We set a random seed, ensuring consistency of results, then retained cross-validation models and predictions. For predictor sets, H2O trained numerous models, generated leader boards ranking model performances, and computed area under the curve (AUC) values. Receiver operating characteristic (ROC) values were visualised as ROC curves, allowing us to compare the performances of the best-in-class models for each predictor set, with diagnostic plots to explain results from the top-performing models.

## Results

**Fig. 1:**
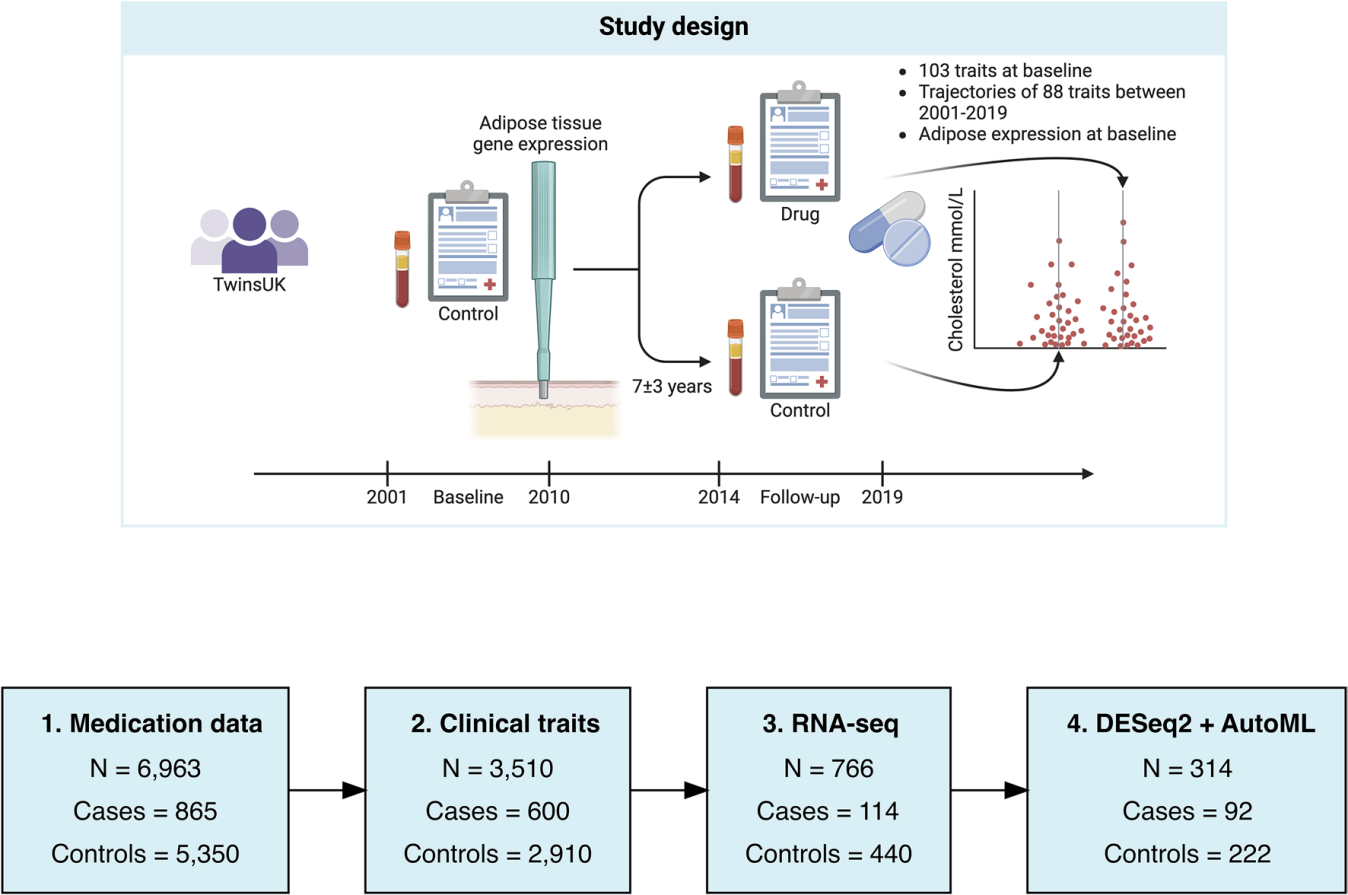
Study design. Participants repeatedly responded to questionnaires which detailed their prescribed medications. We used these responses to identify all participants not using lipid-regulating drugs between 2001–2010 (‘baseline’) then split these participants into cases and controls based on whether they reported taking prescribed lipid-regulating drugs from 2014–2019 (‘follow-up’). A subset of participants (*n* = 766) had transcriptomic data profiled from punch biopsies which included adipose tissue from this baseline period. We compared a catalogue of 103 clinical traits at baseline, 88 trajectories in these traits between baseline and follow-up, and gene expression from adipose tissue samples at baseline for future users of lipid-regulating drug versus controls. Figure adapted from “Double-Blinded, Randomized, Placebo-Controlled, Crossover Study (Graphical, 1)” by BioRender.com (2020). Retrieved from these templates: https://app.biorender.com/biorender-templates.

Using a cohort characterised during routine clinical visits, we investigated the links between 103 clinical traits, molecular markers in adipose tissue, and future lipid-regulating drug use, and their potential as biomarkers of future lipid dysregulation and CVD risk. To achieve this, we obtained medication data between 2 time points: 2001–2010 (‘baseline’) and 2014–2019 (‘follow-up’) (Supplementary Table 1). We established three groups: 5350 participants that were not prescribed drugs at either time point (controls), 865 participants that moved from non-prescribed to prescribed between baseline and follow-up (cases), and 748 participants prescribed drugs throughout (censored). For the 3510 participants with clinical data at both baseline and follow-up (time between visits was 7 ± 3 years on average), covering 4–19 years, we studied how these traits relate to future lipid-regulating drug usage. Then, using a subset of participants with RNA-seq data (*n* = 766), we studied the association between clinical traits and adipose tissue gene expression. Finally, we compared the difference in gene expression between cases and controls, and if this data could predict future lipid-regulating usage using one twin per family (*n* = 312) (Fig. 1; Supplementary Table 3).

### Cardiometabolic and obesity-related traits at baseline increase the risk of future lipid-regulating drug usage

**Fig. 2:**
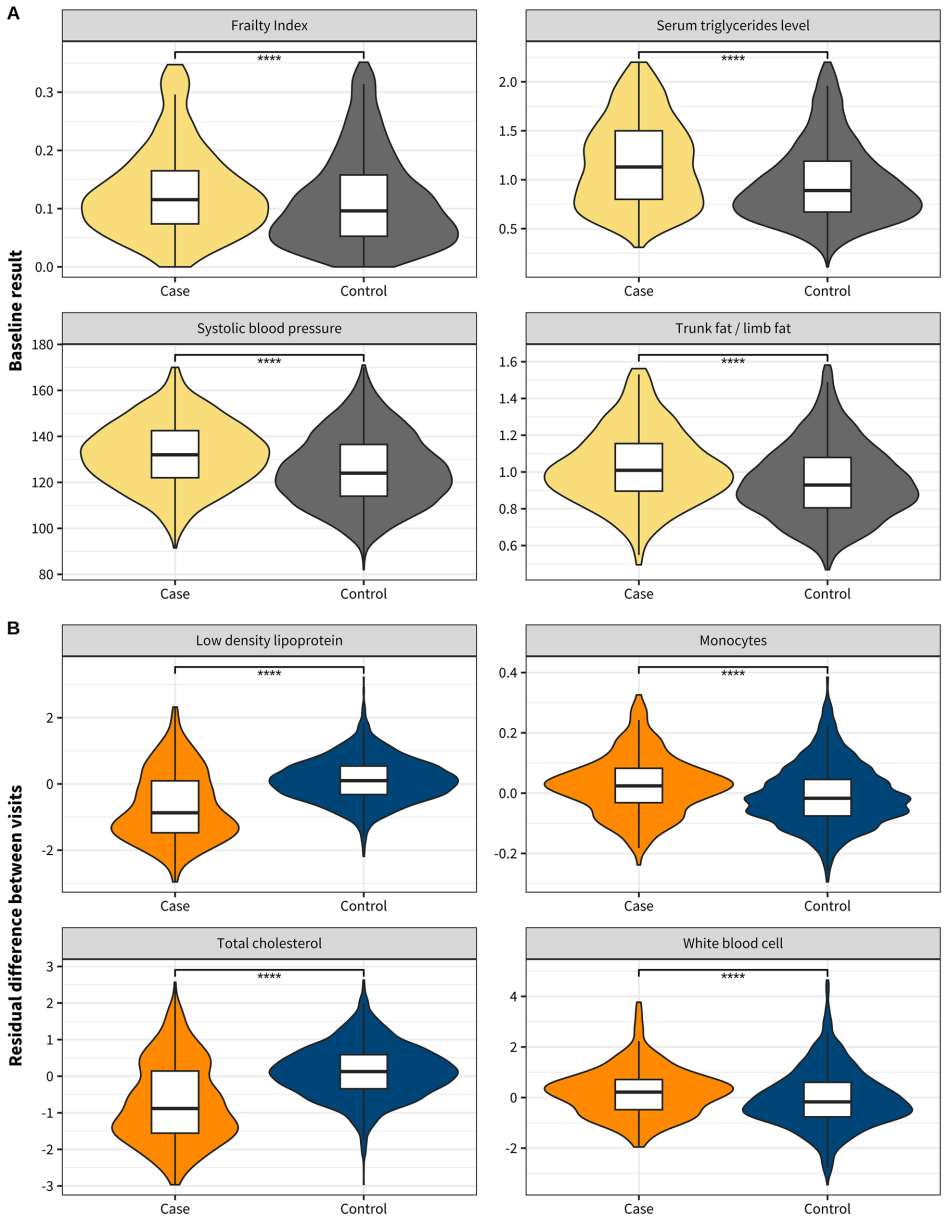
The traits associated with future lipid-regulating drug usage. (**A**) Example of 4 traits at baseline that were associated with future lipid-regulating drug use, which suggest frailty, lipid measures, cardiovascular dysfunction, and abdominal adiposity, are significantly higher in future lipid-regulating drug users at baseline (2001–2010) at least 4 years before reported drug usage. (**B**) Example of changes in 4 traits associated with future lipid-regulating drug use, reflecting decreasing lipid measurements, and increasing immune cell counts, which suggest increased change between visits in future lipid-regulating drug users. Significant differences between cases and controls are indicated by ‘****’ (*p* < 0.0001, Student’s t-test; controls in grey/blue; future medication users in yellow/orange).

To identify the clinical traits associated with future lipid-regulating drug use, we compared the baseline values of 103 clinical traits (e.g., cholesterol, DXA) in those not prescribed drugs at either timepoint (‘controls’) and those who changed from not prescribed drugs at baseline to taking lipid regulating drugs at follow-up (‘cases’). In total, 28 traits produced associations with future lipid-regulating drug use (FDR 5%; Table 1), the most statistically significant of which suggested elevated lipid markers (apolipoprotein B, LDL cholesterol, total cholesterol, and triglycerides in serum; Table 1) increase the likelihood of needing lipid-regulating drugs in the future. These associations are not surprising since we expect individuals with elevated lipid levels to be prescribed lipid-regulating drugs. We found multiple cardiometabolic traits were associated with an increased likelihood of future lipid-regulating drug usage, including ASCVD risk scores, fat in the abdominal region, blood pressure, frailty, and heart rate while HDL cholesterol, heart activity measures, lean tissue and mass in limbs, pancreatic amylase, and creatinine were associated with decreased likelihood of future lipid-regulating drug use (Fig. 2A). Overall, the 28 clinical traits significantly associated with increased likelihood of future lipid-regulating drug use grouped into phenotypes implicating elevated lipid markers, excessive fat tissue the abdominal region, and cardiovascular dysfunction.

**Table 1:** Clinical trait and future lipid-regulating drug usage associations.

Clinical trait	Cases	Controls	Beta	SE	*P*-value
**Age-related**					
Frailty Index	396	1914	0.55	0.16	3.43 × 10^−4^
Nuclear cataracts score	214	873	0.35	0.23	5.63 × 10^−3^
**Cardiovascular**					
ASCVD	409	1997	1.73	0.53	1.69 × 10^−7^
Diastolic blood pressure	573	2916	0.20	0.53	8.25 × 10^−3^
ECG P axis	234	1253	−0.56	0.61	4.87 × 10^−3^
Pulse pressure	562	2907	0.27	0.63	3.95 × 10^−4^
Carotid-femoral pulse wave velocity	172	866	0.41	0.16	6.10 × 10^−3^
Carotid-femoral pulse time	174	869	−0.62	0.33	7.21 × 10^−4^
Systolic blood pressure	567	2916	0.36	0.41	2.75 × 10^−6^
**Dual x-ray absorptiometry**					
All tissue in gynoid region	476	2456	−0.34	0.24	1.39 × 10^−2^
Area of fat inside abdominal cavity	481	2496	0.58	0.20	3.45 × 10^−6^
Fat tissue in trunk region	494	2540	0.63	0.22	7.78 × 10^−5^
Lean tissue in right leg region	490	2505	−0.31	0.27	8.32 × 10^−3^
Mass in right leg region	487	2489	−0.40	0.24	1.67 × 10^−3^
% of fat in android region	488	2506	0.45	0.20	1.46 × 10^−4^
% of fat in visceral fat region	488	2503	0.43	0.20	2.37 × 10^−4^
% of fat in visceral/gynoid region	484	2487	0.38	0.19	3.91 × 10^−5^
% of fat tissue in trunk region	497	2537	0.59	0.21	9.29 × 10^−6^
% of total body mass that is fat	488	2492	0.34	0.20	1.32 × 10^−2^
Trunk fat/limb fat	482	2494	0.45	0.20	5.54 × 10^−8^
**Lipid profiles**					
Apolipoprotein B in Serum	360	1466	1.00	0.11	1.31 × 10^−29^
High density lipoprotein	419	2051	−0.35	0.38	2.36 × 10^−4^
Low density lipoprotein	413	2054	0.89	0.24	1.00 × 10^−23^
Serum triglycerides level	523	2763	0.62	0.24	2.85 × 10^−16^
Total cholesterol	415	2060	0.90	0.22	1.97 × 10^−24^
**Metabolic**					
Pancreatic amylase in serum	176	924	−0.37	0.42	5.13 × 10^−3^
Serum creatinine level	137	863	−0.99	0.97	5.62 × 10^−3^
Total amylase in serum	173	935	−0.34	0.52	1.27 × 10^−2^

Next, we examined if the trajectories of clinical traits between baseline and follow-up were associated with lipid-regulating drug use at follow-up by calculating rate of change between both visits for 88 traits with multiple measurements to see if we observed any differences between cases and controls. Overall, we found 16 clinical trait trajectories were associated with future lipid-regulating drug use (FDR 5%; Table 2). Notably, 12 clinical traits trajectories were increasing and associated with increased likelihood of future lipid-regulating drug use including body fat measures, weight, and monocyte counts. Together, these results suggest larger increases body fat distribution and fat tissue, and immune system markers are linked with lipid-regulating drug use in the future (Fig. 2B). The most significant associations were for lipid markers: LDL cholesterol (*p* = 9.37 × 10^−78^), non-HDL cholesterol (*p* = 6.83 × 10^−22^), and total cholesterol (*p* = 1.18 × 10^−74^), showing decreases between visits increasing the log-odds of future lipid-regulating drug use, which we believe is due to treatment (Table 2). Similarly, declining ASCVD risk scores associated with increased likelihood of lipid-regulating drug use, probably due to declining lipid concentrations and concomitant anti-hypertension drug use lowering future ASCVD risk.

**Table 2:** Clinical trait trajectory and lipid-regulating drug usage associations.

Clinical trait	Cases	Controls	Beta	SE	*P*-value
**Cardiovascular**					
ASCVD	356	1836	−0.40	0.35	8.65 × 10^−6^
**Dual x-ray absorptiometry**					
Body Mass Index	557	2968	0.25	1.23	5.80 × 10^−4^
Area of fat inside abdominal cavity	466	2346	0.22	0.22	2.06 × 10^−3^
Fat tissue in left arm region	465	2326	0.30	0.25	1.11 × 10^−4^
Fat tissue in trunk region	470	2347	0.21	0.25	7.22 × 10^−3^
Lean tissue in left arm region	443	2248	0.23	0.44	8.00 × 10^−3^
% of fat in android region	478	2362	0.24	0.23	2.67 × 10^−3^
% of fat in visceral fat region	479	2360	0.26	0.24	1.10 × 10^−3^
% of fat in visceral/android region	466	2179	0.20	0.24	7.87 × 10^−3^
% of fat in visceral/gynoid region	470	2334	0.30	0.29	1.66 × 10^−4^
Total mass in summary	464	2326	0.23	0.26	2.51 × 10^−3^
Weight	553	2967	0.25	1.27	1.40 × 10^−3^
**Immune-related**					
Monocytes	234	1025	0.33	0.29	2.70 × 10^−3^
**Lipid profiles**					
Low density lipoprotein	437	2064	−2.61	0.59	9.37 × 10^−78^
Non-HDL cholesterol	185	816	−1.11	0.31	6.83 × 10^−22^
Total cholesterol	439	2070	−2.50	0.003	1.18 × 10^−74^

### Molecular processes in adipose tissue are linked with phenotypes that precede lipid-regulating drug usage

To study the relationships between molecular processes in adipose tissue and clinical traits associated with future lipid-regulating drugs usage (28 traits at baseline and 16 trajectories, comprising a total of 44 traits), we investigated associations between these phenotypes and transcriptional profiles in adipose tissue measured at baseline in a subset of 766 twins with available transcriptomic data. The sample size of each comparison ranged between 152 and 760 depending on the availability of clinical data.

**Fig. 3:**
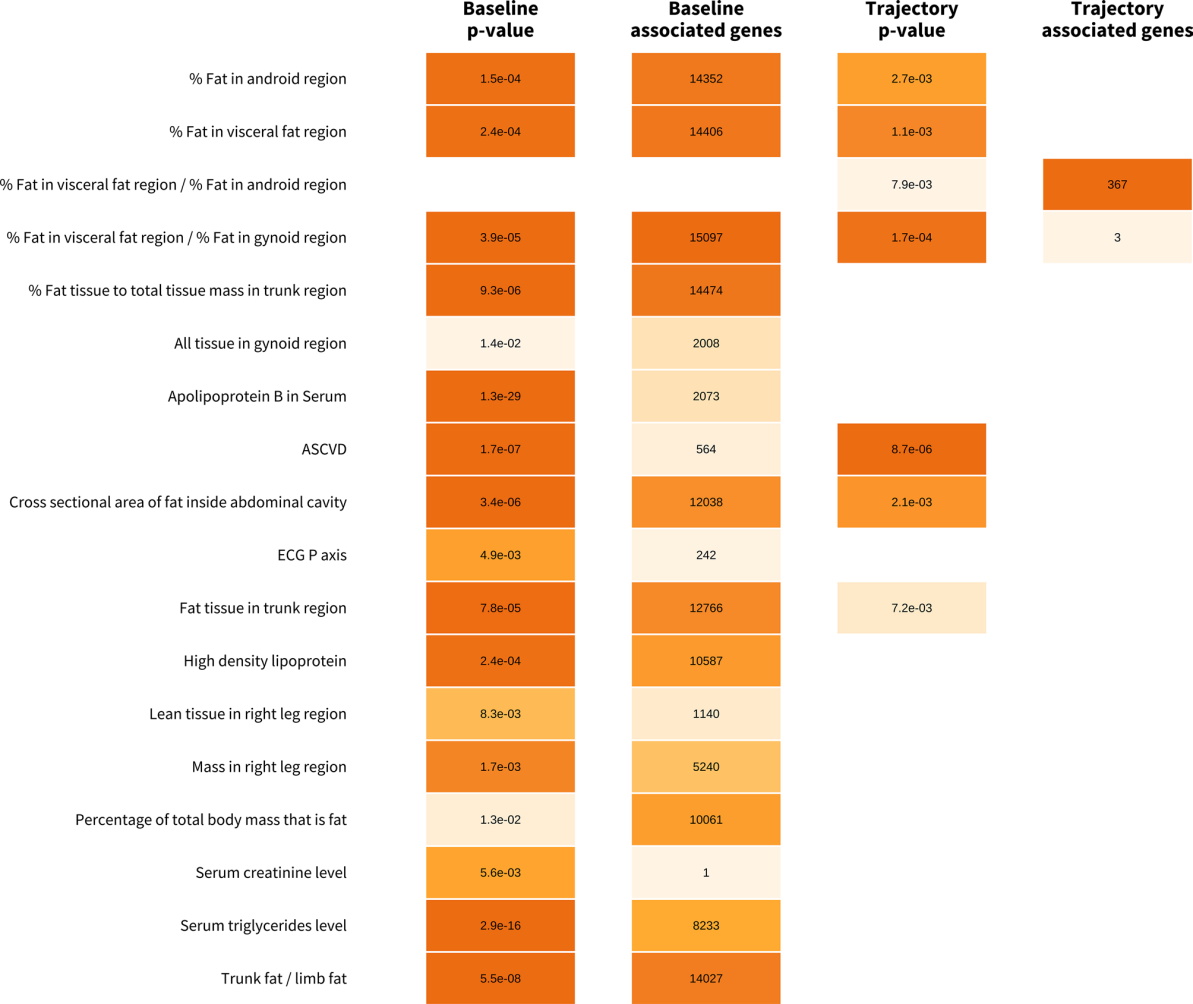
Adipose gene expression associations in traits preceding lipid-regulating drug usage. A p-value heatmap of 19 traits and trajectories associated with future lipid-regulating drugs and the number of genes these were associated with. List only contains clinical traits that were associated with adipose tissue gene expression and future lipid-regulating drug usage below FDR 5%.

17 clinical traits were linked with adipose tissue gene expression at an FDR threshold of 5% including 8 fat tissue measures in the trunk region associated with more than 10,000 genes (Fig. 3). We also identified widespread transcriptome associations between traits reflecting cardiovascular function and health such as the ASCVD risk score (564 associated genes) and ECG P axis (240 associated genes). The ECG P axis represents the direction of depolarisation in the atrium during the cardiac cycle and deviations can indicate elevated risk of arrhythmias or structural heart disease^36^. Notably, several genes associated with ECG P axis (*n* = 242) were enriched for molecular processes implicated in dilated cardiomyopathy (*p* = 7.46 × 10^−4^) and hypertrophic cardiomyopathy (*p* = 1.78 × 10^−3^), while we showed strong enrichment of genes for the ASCVD associated genes with ribosome function (*p* = 1.90 × 10^−41^). These findings show the clinical traits that reflect abdominal fat distribution and cardiovascular functioning both precede lipid-regulating drug use and enrich for molecular processes in adipose.

We revealed associations between baseline adipose gene expression and the trajectories of visceral fat to both gynoid and android fat ratios (3 and 367 associated genes, respectively). The trajectory of the visceral fat to gynoid fat ratio was associated with decreased expression of *RPL7* (*p* = 1.25 × 10^−6^) and *NONO* (*p* = 4.08 × 10^−6^), a member of the 60 S ribosomal subunit^37^ and RNA-binding protein involved in transcriptional regulation through RNA splicing^38^, and increased expression of *LRRC32* (*p* = 8.71 × 10^−6^), a type-I membrane protein. 367 genes were associated with the change in visceral fat to android fat ratio and functionally enriched for terms related to the mitochondria including the mitochondrial matrix (*p* = 7.92 × 10^−14^), Krebs cycle (*p* = 1.86 × 10^−8^), fatty acid oxidation (*p* = 2.07 × 10^−6^), mitophagy (*p* = 1.72 × 10^−2^), and NF-kappa b signalling (*p* = 2.59 × 10^−2^). In addition, these associated genes also enriched for molecular processes associated with mortality, ageing, and age of death (*p* = 5.18 × 10^−3^). We compared the 367 genes with 2,139 age-associated genes in adipose tissue reported by the METSIM study^39^ and found an overlap of 32 genes (8.7%), indicating that some genes associated with this clinical trait have expression levels that are also associated with ageing. We also performed sensitivity analyses to assess robustness of our results to heterogeneity in cell-type or socioeconomic status and found significant effect size correlations after including these covariates in our models in all cases (mean *R* = 0.93 and 0.96, respectively). The full gene lists and corresponding beta estimates and p-values for the clinical traits that are associated with adipose tissue gene expression can be found in Supplementary Table 5. Overall, these results suggest that clinical trait associated with future lipid-regulating drug use overlap with processes in adipose tissue implicating age-related pathways.

### Angiogenesis genes are differentially expressed years before medication use

**Fig. 4:**
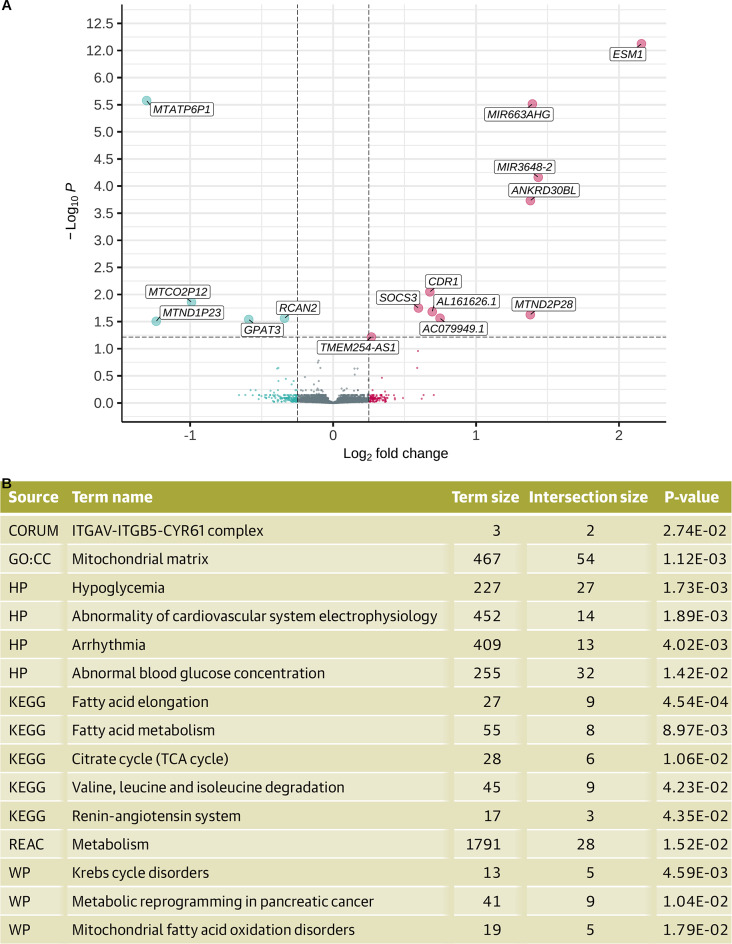
Differential expression between future lipid-regulating drug users versus controls. (**A**) Volcano plot of log-fold change (LFC) and adjusted p-values for future lipid-regulating drug users versus controls with downregulated (LFC < −0.25) and upregulated (LFC > 0.25) genes shown in green and purple, respectively. (**B**) Gene ontology for functional enrichment analysis of 1212 differentially expressed genes (*p* < 0.05) between future lipid-regulating drug users and controls with their respective sources, terms, term sizes, intersection size, and p-values. Functional enrichment table adapted from gprofiler output.

To identify markers of future lipid-regulating drug usage, we investigated genes which were differentially expressed in adipose tissue at baseline of cases (*n* = 92) compared with controls (*n* = 222). We revealed 15 differentially expressed genes below FDR 10%: 10 upregulated and 5 downregulated. The most statistically significant finding was *ESM1* (log_2_-fold change = 2.16, *p* = 3.63 × 10^−17^), which was more highly expressed in the adipose tissue of participants that were prescribed lipid-regulating drugs in the future versus controls. *ESM1* is expressed by endothelial cells and tightly regulated by inflammatory cytokines (TNF-α and IFN-γ) and has transcript isoforms that increase with pro-angiogenic factors^40^. In addition, *ANKRD30BL*, *SOCS3*, and *TMEM254-AS1*, and microRNAs *MIR3648-2* and *MI663AHG* were upregulated, while *RCAN2* and *GPAT3* were downregulated in future lipid-regulating drug users (Fig. 4A). Three of four significant mitochondrial pseudo-genes (*MTATP6P1*, *MTCO2P12*, *MTND2P28*, and *MTND1P23*) were downregulated^41^. Then, functional enrichment of the 1212 genes differentially expressed below the nominal p-value threshold (*p* < 0.05; 538 upregulated and 674 downregulated) showed processes related to the mitochondrial matrix (*p* = 1.12 × 10^−3^), abnormal cardiovascular system electrophysiology (*p* = 1.89 × 10^−3^), and arrhythmia (*p* = 4.02 × 10^−3^; Fig. 4B). The complete gene ontology and pathway enrichment results from the genes differentially expressed in future lipid-regulating drug users is in Supplementary Tables 6 and the frequency of genes across the gene ontology terms is shown in Supplementary Table 7. In addition, we identified 1,116 genes at nominal significance with a limma-voom approach. From these, 840 genes were common to both methods (Supplementary Table 8), comprising a true-positive rate of 0.753 and false-positive rate of 0.019 when using limma-voom results as the reference. The log_2_-fold change estimates produced a strong correlation (*R* = 0.82, *p* = 2.2 × 10^−16^), indicating our findings are robust in multiple analytical frameworks (Supplementary Fig. 1). Lastly, to assess if individuals who go on to need lipid-regulating drugs showed evidence of accelerated molecular ageing in adipose tissue, we tested deviations in epigenetic age from chronological age in 510 participants with available adipose-derived 450K methylation array data (cases = 76; controls = 347)^19^. While cases had a higher mean age acceleration (mean = 0.138) versus controls (mean = 0.004), the differences between cases and controls were not statistically significant when tested with Welch’s two sample t-test (*p* = 0.3). Though, adding adipose tissue epigenetic age acceleration estimates to mixed models of future drug usage significantly improved model fit (∆AIC = −17, LRT *p* = 9.9 × 10^−6^), where a 1 SD increase in age acceleration had an odds ratio of 1.75, indicating 75% higher odds of future drug use. Thus, accelerated molecular ageing in adipose tissue may precede future lipid-regulating drug use. Overall, our results suggest that cell signalling pathways may differ in individuals who go on to need lipid-regulating drugs around five years before reporting any treatment and appear consistent with molecular processes enriched in traits preceding medication use. However, future studies should explore if other factors including cohort composition and sample size are important in producing the expression differences observed in our study.

### Genes significantly associated with clinical traits exhibit larger differences when comparing cases and controls

In addition, we explored if the genes that were associated with a relevant clinical trait also exhibited increased evidence of differential expression between cases and controls. For each of the 18 clinical traits associated with future drug use, we compared the log_2_-fold changes from the case-versus-control differential expression analysis for the set of genes associated with that trait (FDR threshold 5%) versus genes not associated with the trait. We discovered significant differences in 16 traits (Table 3): 12 DXA traits (including fat and lean mass traits), 3 lipid traits, and ECG P axis, with large effects seen for apolipoprotein B (*p* = 1.01 × 10^−127^), triglycerides (*p* = 1.55 × 10^−29^), HDL (*p* = 2.74 × 10^−8^), and ECG measurements (*p* = 2.19 × 10^−4^). These findings suggest the shared molecular processes that underlie all these clinical traits are more active at baseline in individuals prescribed lipid-lowering medication in the future. We found 23 genes associated with 14/16 clinical traits implicating mitochondrial processes (*BNIP3L*, *HADH*, *MTARC2*)^42–48^, apoptosis (*E2F1*, *GSDMB*)^49,50^, cardiovascular function (*GPD1L*, *PDK2*, *TBX4*)^51–59^, immunity (*IL1RN*)^60^, and lipid metabolism (*SLC27A2*)^61^. Notably, *TBX4*, from the T-box gene family, which serves a crucial role during development, has been connected to BMI and waist-hip ratio phenotypes in GWAS meta-analysis studies, and various *TBX* genes are implicated in both adipogenesis and the browning of adipocytes^62–66^. Additionally, *GPAT3* was a gene which showed larger log_2_-fold changes for 14 out of 16 clinical traits as well as producing differential expression in cases. *GPAT3* protects against lipo-toxicity in cells^67^ and was downregulated, which might suggest that individuals with lower expression show more lipid accumulation in non-adipose tissues. Finally, we enriched genes associated with each of the 16 phenotypes against CORUM, HP, GO, KEGG, and REACTOME and combined terms to see which were most common. The most common molecular terms in 12 out of 16 traits related to abnormalities in metabolite concentrations and homeostasis, and hypoglycaemia, and neutrophil degranulation^68^. In conclusion, these results suggest traits that precede future lipid-regulating drug use share common molecular mechanisms in adipose tissue, implicating widespread metabolic dysregulation.

**Table 3:** Comparison of log2-fold change between cases and controls for genes associated with clinical traits preceding lipid-regulating drug usage.

Phenotype	FDR > 5%	FDR < 5%	∆ LFC	*P*-value
**Cardiovascular**				
ECG P axis	20,171	242	0.0074	2.19 × 10^−4^
**Dual x-ray absorptiometry**				
All tissue in gynoid region	18,414	1999	0.0066	6.77 × 10^−18^
Area of fat inside abdominal cavity	8431	11,982	0.0040	3.25 × 10^−22^
Fat tissue in trunk region	7712	12,701	0.0022	9.84 × 10^−8^
Lean tissue in right leg region	19,276	1137	0.0084	4.32 × 10^−17^
Mass in right leg region	15,197	5216	0.0060	4.56 × 10^−33^
% of fat in android region	6153	14,260	0.0017	7.68 × 10^−5^
% of fat in visceral region	6097	14,316	0.0015	3.51 × 10^−4^
% of fat in visceral/gynoid region	5423	14,990	0.0009	2.18 × 10^−2^
% of fat tissue in trunk region	6036	14,377	0.0020	2.73 × 10^−6^
% of total body mass that is fat	10,385	10,028	0.0028	6.08 × 10^−12^
Trunk fat	6485	13,928	0.0016	1.88 × 10^−4^
∆ % of fat in visceral/android region	20,046	367	0.019	8.22 × 10^−21^
**Lipid profiles**				
Apolipoprotein B	18,343	2070	0.022	1.01 × 10^−127^
High density lipoprotein	9883	10,530	0.0022	2.74 × 10^−8^
Serum triglycerides level	12,214	8199	0.0049	1.55 × 10^−29^

### Predicting future lipid-regulating drug users

To determine the utility of baseline clinical phenotypes and adipose tissue gene expression for predicting future lipid-regulating drug usage, we leveraged automated machine learning to combine numerous features into predictor sets and optimise their predictive power with 100 machine learning algorithms comprising regression models, gradient boosted machines, and stacked ensembles. We investigated 5 combinations of features: (1) the ASCVD risk scores, (2) values of the 28 clinical traits associated with future lipid-regulating drug use in the study, (3) gene expression values associated with future lipid-regulating drug usage below FDR of 5% (*n* = 15), (4) adipose gene expression values associated with future lipid-regulating drug usage at a relaxed threshold of *p* < 0.05 (*n* = 1212 genes), and (5) clinical traits including ASCVD risk and adipose gene expression that passed the nominal threshold of *p* < 0.05 (*n* = 1212 genes). We selected the leading model based on AUC for each feature combination (Table 4).

**Table 4:** Summary of model performance predicting future lipid-regulating drug use.

Combination	Features	AUC
1	ASCVD	0.722
2	28 Traits including ASCVD (FDR 5%)	0.844
3	15 Genes (FDR 5%)	0.651
4	1212 Genes (*p* < 0.05)	0.904
5	28 Traits + 1212 Genes	0.919

**Fig. 5:**
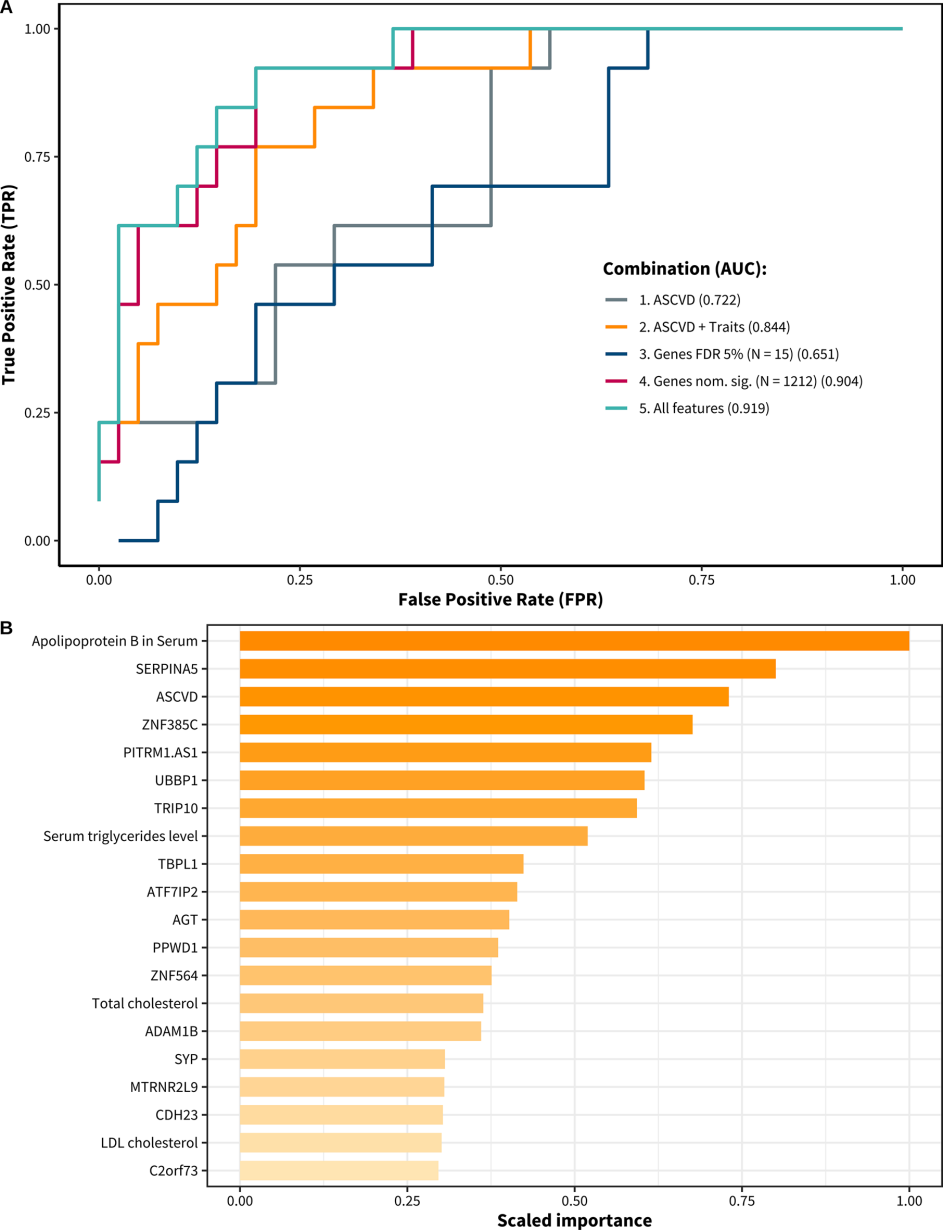
Evaluating model performance predicting future lipid-regulating drug usage with five different combinations of 28 associated clinical traits and adipose tissue gene expression. (**A**) Receiver Operating Characteristic (ROC) curves for five predictive models that incorporate various combinations of clinical phenotypes and gene expression for predicting future use of lipid-regulating drugs. Each curve represents one of the combinations outlined in Table 4, with the x-axis reflecting the false positive rate and the y-axis showing the true positive rate. The area under the curve (AUC) value corresponds to the models’ performance, illustrating the trade-offs between sensitivity and specificity across different thresholds. A curve closer to the top-left corner signifies a better-performing model, as evidenced by the highest AUC value associated with the combination of 28 traits and 1212 genes (AUC = 0.919), suggesting this model exhibited the greatest capacity to discriminate future lipid-regulating drug users. (**B**) Variable importance plot for the XGBoost model that achieved the highest AUC. The x-axis lists the variables, which includes clinical phenotypes such as apolipoprotein B in Serum and ASCVD risk, and the expression of genes such as SERPINA5 and UBBP1. The x-axis quantifies the importance scores, reflecting the extent and frequency to which each variable improves the model’s overall performance.

We observed a wide range of performance for the 5 combinations, ranging from an AUC of 0.651 to 0.919. The best model for the ASCVD risk score demonstrated modest performance (AUC = 0.722), highlighting the limitations of relying only on clinical risk scores (Table 4). Introducing the associated clinical phenotypes led to a significant improvement. For example, the combination of 28 associated clinical traits including ASCVD risk scores (Combination 2) achieved a markedly enhanced AUC of 0.844, highlighting the utility of baseline clinical trait information to improve predictions. Overall, Combination 5 returned the best performance, which integrated 1212 nominally significant gene expression values and clinical phenotypes including ASCVD risk, with the improved AUC of 0.919 (Fig. 5A). While the expression values of the 15 genes passing an FDR of 5% in isolation produced poor predictions (AUC = 0.651), using expression values of the 1212 genes with nominal significance produced a model with an impressive predictive capacity (AUC = 0.904), outperforming the ASCVD risk score alone and combined with clinical traits, and nearly matching the maximum AUC of 0.919 observed in Combination 5. These findings suggest that in the absence of detailed epidemiological or clinical data, baseline molecular signatures from adipose tissue could be features for predicting future lipid-regulating drugs usage beyond traditional risk scores.

To establish the baseline features within our best performing combination (Combination 5) that were most important, we examined the contributions and importance of these features to this model using several strategies. Variable importance analysis calculates the influence of variables in the prediction model by calculating the change in performance when features are excluded; this analysis demonstrated that the lipid markers including ASCVD risk scores apolipoprotein B, and serum triglycerides levels were highly important, and the remaining most important variables corresponded to gene expression values, emphasising the role of these molecular signatures in improving predictive power within this combination (Fig. 5B, also validated by model correlations heatmaps, Supplementary Fig. 2 A). Furthermore, SHAP (Shapley additive explanations) analysis, which tries to quantify the relative contributions of features in the model for each participant, further supported the role of gene expression in delivering accurate predictions for this combination (Supplementary Fig. 2B). These results indicate that the top-performing model represents risk of future lipid-regulating drug use by combining complex gene expression patterns in adipose tissue and traditional lipid markers. Overall, these results suggest that molecular signatures in adipose tissue can improve upon traditional risk assessments when identifying individuals at risk for clinical health outcomes, especially when combined with clinical traits.

## Discussion

Here, we drew on longitudinal clinical data and adipose tissue gene expression to implicate specific clinical traits and molecular processes in future lipid-regulating drug use. We found robust associations between 28 traits at baseline and trajectories of 16 traits over time with future lipid-regulating drug use, which demonstrated increased cardiovascular, metabolic, and obesity markers precede lipid-regulating drug use. We revealed relationships between these 44 phenotypes and molecular processes in adipose tissue, identifying 19 phenotypes (17 traits and 2 trajectories) that associated with adipose tissue gene expression at baseline. We further identified 15 genes that were differentially expressed years before prescription of lipid-regulating drugs and these highlighted molecular processes including immunity and respiratory processes, which were altered several years before prescription.

We found many DXA measures of body fat distribution, and their trajectories, are associated with the use of lipid-regulating drugs. Notably, we demonstrated how the rate of change in the size and composition of fat tissue in the abdomen is strongly associated with medication use at follow-up visits and molecular processes in adipose tissue. For example, the ratio of visceral fat to android fat alone was not strongly associated with future drug use at baseline, but the change in this trait over time was, and was also associated with molecular processes in adipose tissue. However, caution is warranted when interpreting these results as this link could suggest that alterations in body fat distribution in these areas increases the likelihood of lipid treatment, or that both lipid treatment and increasing fat deposits in these areas are correlated with another mechanism. More research is needed to contextualise these findings. Interestingly though, we observed common molecular processes enriched with these genes and genes differentially expressed between future lipid-regulating drug users and controls. These included the Krebs cycle^69^, and the citrate shuttle^70^. Importantly, dysregulation of these processes can lead to mitochondrial dysfunction and impaired ATP generation^71^, which lead to the accelerated development of cardiovascular disease^72^. The accumulation of visceral fat has been linked to mitochondrial dysfunction in adipocytes, which can impair energy metabolism and exacerbate CVD risk factors such as atherosclerosis and hypertension^73^. Visceral fat is highly metabolically active and linked with the release of pro-inflammatory cytokines^74^, such as IL-6 and TNF-a, which were implicated in many of the molecular and cellular pathways enriched here and interact with many of the genes that were also differentially expressed in future lipid-regulating drug users at baseline [see below]. Their pro-inflammatory influence is additionally compounded by the secretion of fatty acids into circulation, which promote dyslipidaemia^75^, linking adiposity in the form of visceral fat accumulation with dysregulated lipid metabolism and cardiometabolic disease risk.

We detected 15 genes that were differentially expressed between cases and controls years before participants reported taking any lipid-lowering medication. Many of these genes are well-characterised with respect to their roles within hallmarks of cellular ageing^76^. Angiogenesis is a biological stress response and repair mechanism after ischemic injury^77^. The most significant gene *ESM1* is linked with cardiomyopathies and myocardial infarctions^78–81^ and proven to promote angiogenic sprouting and interact with leukocytes through TNF-a and IL1-b^40^, hence expression for this gene might be upregulated in adipose tissue in response to damage of adjacent vasculature. *RCAN2* variants are also related to BMI and cellular pathways in cardiac hypertrophy and immune response in lymphocytes. Moreover, *RCAN2* gene expression in endothelial cells has been discovered to function downstream of transcriptional factors which induce angiogenesis^82–84^. *SOCS3* has been observed during angiogenesis as a negative regulator from studies of both human small cell lung cancer and colorectal cancers, while dysregulation of *SOCS3* gene expression has been associated with both CAD and related conditions including acute coronary syndrome^85–88^. Furthermore, MicroRNAs have been increasingly implicated as markers for cardiometabolic diseases^89^. MicroRNA-663 is a marker for pulmonary arterial hypertension, which prevents hypertrophy through TGF-β1/smad2/3^90^. It regulates processes important in atherosclerosis including smooth muscle cell phenotypic switch, vascular neointimal formation, and stress responses to oxidised phospholipids in endothelial cells^91,92^. MicroRNA 3648-1 is also upregulated after endoplasmic reticulum stress and loss of proteostasis^93^. We attempted to compare nominal hits from this analysis with results from similar studies in other tissues, such as liver^94^, and found no evidence of overlap (4 out of 98 genes), however, most of these studies were not directly comparable as they focused on gene expression differences after drug use (response) rather than before (risk), or did not use healthy individuals (e.g., severely obese). Since subcutaneous adipose tissue plays a critical role in fatty acid metabolism and secretes adipokines regulating inflammation, insulin sensitivity, and lipid homeostasis^95^, our study suggests that individuals at increased risk of future lipid-regulating drug use are presenting early signs of immune disruption, lipid dysregulation, or vascular remodelling in this tissue, which impacts its primary endocrine and metabolic functions, leading to perturbations that intersect with numerous age-related molecular phenotypes^96^.

Together, the results presented here identify a variety of molecular markers and processes which influence CVD phenotypes and may prove valuable as prognostic markers of CVD risk and improve personalised medical interventions. From investigating the implementation of these features within automated machine learning algorithms, we have demonstrated that combining clinical traits with gene expression holds the potential to enhance risk prediction. Adipose gene expression values had impressive predictive ability, which both outperformed the predictive performance of the ASCVD risk score and achieved best-in-class performance when combined with clinical markers. The dramatic increase in predictive power seen when incorporating expression of genes at more permissive multiple testing thresholds (from AUC 0.651–0.904) suggests interactions between these genes holds a stronger predictive capacity than the top associated genes. Encouragingly, these models could be improved further still with larger sample sizes and better-quality data, and would afford greater statistical power, better representation of the underlying population, greater tolerance to noise and outliers, and reduced bias, leading to more reliable and accurate predictions, and thereby enhancing early detection of disease.

The results described are limited in several ways. The definition of lipid dysregulation relies on reported medication use and there are confounding factors which influence the accuracy of this as a proxy for physiology. Lipid-regulating drugs are prescribed based on a diagnosis by a medical professional, but the biological criteria for prescription can vary. For example, lipid-regulating drugs may be prescribed prospectively based on family history or risk factors before lipid dysregulation has occurred in some individuals. Participants may be inaccurate during their self-reporting, and we cannot tell precisely when participants were first put on lipid-regulating drugs as data was collected at intervals of two years. Nevertheless, we tried to account for this by utilising 4-year windows between questionnaire responses but cannot account for the precise time participants were prescribed drugs. Moving forward, we could run these analyses again with electronic health record data and increase the result accuracy by fully accounting for the time between baseline measurement and future medication use. We also used crude measures of the rate of change for clinical traits between visits. Though, despite the lack of prescription dates and varying follow-up times, we were able to produce models that significantly outperformed traditional risk assessment. Lastly, due to the known associations with our outcome, it is possible that our method of feature selection could lead to data leakage and inflation of the absolute AUC values for our predictive models, however, this should not compromise the relative performance of each model combination compared to another, which highlighted an improved performance when including gene expression and clinical traits compared to traditional clinical risk prediction scores in isolation. Additionally, our gene-level observations will be sensitive to cohort composition factors like sample size and should be regarded as hypothesis-generating until validated in an independent dataset. Our findings are based solely on the TwinsUK cohort, which comprises British individuals of mostly European-ancestry and is primarily female (82%). As a result, our ability to generalise findings to more diverse ancestral groups or sex-balanced populations is undetermined.

Overall, the findings of this study suggest some people may benefit from being prescribed lipid-regulating drugs several years before dysregulated lipid metabolism is identified clinically. Though, pragmatically it may be advisable to optimise lifestyle changes in line with the NICE guidelines, such as increased physical activity and weight management, before relying on medical interventions, and it is possible some of that some of the observed changes over time derive from those participants who opted for non-pharmacological routes to reduce their disease risk. Similarly, it is important to acknowledge that while adipose tissue offers a powerful resource to uncover the molecular determinants of CVD risk, accessing this tissue on a population level might not be clinically tractable due to its difficulty of extraction. Finally, the integration of genetic markers into these models could offer more understanding of the interplay between genetic predisposition, adipose tissue gene expression, and CVD risk, possibly refining our strategies for pharmacological and non-pharmacological interventions in the future.

## Conclusions

In conclusion, these results highlight robust early phenotypic and transcriptional differences in future lipid-regulating drugs users, implicating molecular processes as early warning signs of dysregulated lipid metabolism. Incorporating these traits as features in predictive models discriminated future lipid-regulating drug users better than traditional risk prediction scores and could be valuable when preventing future cases of CVD related to lipid dysregulation.

## Supplementary Information

Below is the link to the electronic supplementary material.

Supplementary Material 1

Supplementary Material 2

## Data Availability

Data access requests are overseen by the TwinsUK Resource Executive Committee (TREC). For information on access to these genotype and phenotype data and how to apply, see https://twinsuk.ac.uk/researchers/access-data-and-samples/request-access/. The analysis supporting the conclusions of this article are available in the GitHub repository https://github.com/Max-Tomlinson/Lipid-dysregulation.

## Abbreviations

ASCVD: Atherosclerotic cardiovascular disease
AUC: Area under the curve
CAD: Coronary artery disease
CVD: Cardiovascular disease
DXA: Dual x-ray absorptiometry
ECG P: Electrocardiogram P wave
ECG RR: Electrocardiogram RR interval
GWAS: Genome-wide association study
FDR: False discovery rate
HDL: High-density lipoprotein
LDL: Low-density lipoprotein
ML: Machine learning
PCA: Principal component analysis
ROC: Receiver operating characteristic
TMM: Trimmed mean of M-values

## References

[1] O’Donnell, M. J. et al. Global and regional effects of potentially modifiable risk factors associated with acute stroke in 32 countries (INTERSTROKE): a case-control study. *Lancet.* **388** (10046), 761–775 (2016). 27431356 10.1016/S0140-6736(16)30506-2

[2] Libby, P. et al. *Atherosclerosis Nat. Rev. Dis. Primer. ***5 **(1), 56 (2019). 10.1038/s41572-019-0106-z 31420554

[3] Melzer, D., Pilling, L. C. & Ferrucci, L. The genetics of human ageing. *Nat. Rev. Genet.* **21** (2), 88–101 (2020). 31690828 10.1038/s41576-019-0183-6 PMC9934000

[4] Erdmann, J., Kessler, T., Munoz Venegas, L. & Schunkert, H. A decade of genome-wide association studies for coronary artery disease: the challenges ahead. *Cardiovasc. Res.* **114 **(9), 1241–1257 (2018). 10.1093/cvr/cvy084 29617720

[5] Khera, A. V. & Kathiresan, S. Genetics of coronary artery disease: discovery, biology and clinical translation. *Nat. Rev. Genet.* **18** (6), 331–344 (2017). 28286336 10.1038/nrg.2016.160 PMC5935119

[6] González-Muniesa, P. et al. *Obes. Nat. Rev. Dis. Primer. ***3 **(1), 17034 (2017). 10.1038/nrdp.2017.34 28617414

[7] Salvestrini, V., Sell, C. & Lorenzini, A. Obesity May accelerate the aging process. *Front. Endocrinol.* **10**, 266 (2019). 10.3389/fendo.2019.00266 PMC6509231 31130916

[8] Ferrucci, L. & Fabbri, E. Inflammageing: chronic inflammation in ageing, cardiovascular disease, and frailty. *Nat. Rev. Cardiol.* **15** (9), 505–522 (2018). 30065258 10.1038/s41569-018-0064-2 PMC6146930

[9] Libby, P., Ridker, P. M. & Hansson, G. K. Inflammation in atherosclerosis: from pathophysiology to practice. *J. Am. Coll. Cardiol.* **54** (23), 2129–2138 (2009). 19942084 10.1016/j.jacc.2009.09.009 PMC2834169

[10] Kershaw, E. E. & Flier, J. S. Adipose tissue as an endocrine organ. *J. Clin. Endocrinol. Metab.* **89** (6), 2548–2556 (2004). 15181022 10.1210/jc.2004-0395

[11] Oikonomou, E. K. & Antoniades, C. The role of adipose tissue in cardiovascular health and disease. *Nat. Rev. Cardiol.* **16** (2), 83–99 (2019). 30287946 10.1038/s41569-018-0097-6

[12] Cinti, S. et al. Adipocyte death defines macrophage localization and function in adipose tissue of obese mice and humans. *J. Lipid Res.* **46** (11), 2347–2355 (2005). 16150820 10.1194/jlr.M500294-JLR200

[13] González-Muniesa, P. et al. Effects of hyperoxia on Oxygen-Related inflammation with a focus on obesity. *Oxid. Med. Cell. Longev.* **2016**, 8957827 (2016). 10.1155/2016/8957827 PMC4678090 26697142

[14] Robinson, E. L. et al. Dissecting the transcriptome in cardiovascular disease. *Cardiovasc. Res.* **118** (4), 1004–1019 (2022). 33757121 10.1093/cvr/cvab117 PMC8930073

[15] McCarthy, M. & Birney, E. Personalized profiles for disease risk must capture all facets of health. *Nature.* **597** (7875), 175–177 (2021). 34489576 10.1038/d41586-021-02401-0

[16] Verdi, S. et al. TwinsUK: the UK adult twin registry update. *Twin Res. Hum. Genet.* **22** (6), 523–529 (2019). 31526404 10.1017/thg.2019.65

[17] Zhang, X. et al. PPI-Induced changes in plasma metabolite levels influence total hip bone mineral density in a UK cohort. *J. Bone Min. Res. Off J. Am. Soc. Bone Min. Res.* **38** (2), 326–334 (2023). 10.1002/jbmr.4754 PMC10108201 36458982

[18] Doll, H., Shine, B., Kay, J., James, T. & Glasziou, P. The rise of cholesterol testing: how much is unnecessary. *Br. J. Gen. Pract.* **61** (583), 81–88 (2011). 21276328 10.3399/bjgp11X556245 PMC3026174

[19] Christiansen, C. et al. Adipose methylome integrative-omic analyses reveal genetic and dietary metabolic health drivers and insulin resistance classifiers. *Genome Med.* **14** (1), 75 (2022). 35843982 10.1186/s13073-022-01077-z PMC9290282

[20] Searle, S. D., Mitnitski, A., Gahbauer, E. A., Gill, T. M. & Rockwood, K. A standard procedure for creating a frailty index. *BMC Geriatr.* **8** (1), 24 (2008). 18826625 10.1186/1471-2318-8-24 PMC2573877

[21] 2013 ACC/AHA Guideline on the Assessment of Cardiovascular Risk.

[22] Steves, C. J., Jackson, S. H. D. & Spector, T. D. Cognitive change in older women using a computerised battery: A longitudinal quantitative genetic twin study. *Behav. Genet.* **43** (6), 468–479 (2013). 23990175 10.1007/s10519-013-9612-z PMC3825151

[23] Glastonbury, C. A., Couto Alves, A., El-Sayed Moustafa, J. S. & Small, K. S. Cell-Type heterogeneity in adipose tissue is associated with complex traits and reveals Disease-Relevant Cell-Specific eQTLs. *Am. J. Hum. Genet.* **104** (6), 1013–1024 (2019). 31130283 10.1016/j.ajhg.2019.03.025 PMC6556877

[24] Pan, B. et al. Similarities and differences between variants called with human reference genome HG19 or HG38. *BMC Bioinform.* **20** (2), 101 (2019). 10.1186/s12859-019-2620-0 PMC6419332 30871461

[25] Robinson, M. D. & Oshlack, A. A scaling normalization method for differential expression analysis of RNA-seq data. *Genome Biol.* **11** (3), 25 (2010). 20196867 10.1186/gb-2010-11-3-r25 PMC2864565

[26] Lai, Y. A statistical method for the Conservative adjustment of false discovery rate (q-value). *BMC Bioinform.* **18** (3), 69 (2017). 10.1186/s12859-017-1474-6 PMC5374657 28361675

[27] Jafari, M., Ansari-Pour, N. & Why When and how to adjust your P values? *Cell. J. Yakhteh. ***20 **(4), 604-607 (2018). 10.22074/cellj.2019.5992 PMC6099145 30124010

[28] Viñuela, A. et al. Age-dependent changes in mean and variance of gene expression across tissues in a twin cohort. *Hum. Mol. Genet.* **27** (4), 732–741 (2018). 29228364 10.1093/hmg/ddx424 PMC5886097

[29] Ignatiadis, N., Klaus, B., Zaugg, J. B. & Huber, W. Data-driven hypothesis weighting increases detection power in genome-scale multiple testing. *Nat. Methods*. **13** (7), 577–580 (2016). 27240256 10.1038/nmeth.3885 PMC4930141

[30] Love, M. I., Huber, W. & Anders, S. Moderated Estimation of fold change and dispersion for RNA-seq data with DESeq2. *Genome Biol.* **15** (12), 550 (2014). 25516281 10.1186/s13059-014-0550-8 PMC4302049

[31] Chen, X., Zhang, B., Wang, T., Bonni, A. & Zhao, G. Robust principal component analysis for accurate outlier sample detection in RNA-Seq data. *BMC Bioinform.* **21** (1), 269 (2020). 10.1186/s12859-020-03608-0 PMC7324992 32600248

[32] Ritchie, M. E. et al. Limma powers differential expression analyses for RNA-sequencing and microarray studies. *Nucleic Acids Res.* **43** (7), 47 (2015). 25605792 10.1093/nar/gkv007 PMC4402510

[33] Law, C. W., Chen, Y., Shi, W. & Smyth, G. K. Voom: precision weights unlock linear model analysis tools for RNA-seq read counts. *Genome Biol.* **15 **(2), 29 (2014). 10.1186/gb-2014-15-2-r29 PMC4053721 24485249

[34] Horvath, S. DNA methylation age of human tissues and cell types. *Genome Biol.* **14** (10), 115 (2013). 24138928 10.1186/gb-2013-14-10-r115 PMC4015143

[35] Eiriksdottir, T. et al. Predicting the probability of death using proteomics. *Commun. Biol.* **4** (1), 758 (2021). 34145379 10.1038/s42003-021-02289-6 PMC8213855

[36] Dimala, C. A., Nso, N., Wasserlauf, J. & Njei, B. Electrocardiographic abnormalities in patients with metabolic dysfunction-associated steatotic liver disease: A systematic review and meta-analysis. *Curr. Probl. Cardiol.* **49** (7), 102580 (2024). 38653446 10.1016/j.cpcardiol.2024.102580

[37] Odintsova, T. I. et al. Characterization and analysis of posttranslational modifications of the human large cytoplasmic ribosomal subunit proteins by mass spectrometry and Edman sequencing. *J. Protein Chem.* **22** (3), 249–258 (2003). 12962325 10.1023/a:1025068419698

[38] Zhang, Z. & Carmichael, G. G. The fate of DsRNA in the nucleus: A p54nrb-Containing complex mediates the nuclear retention of promiscuously A-to-I edited RNAs. *Cell.* **106** (4), 465–475 (2001). 10.1016/s0092-8674(01)00466-4 11525732

[39] Brotman, S. M. et al. Cell-Type composition affects adipose gene expression associations with cardiometabolic traits. *Diabetes.* **72** (11), 1707–1718 (2023). 37647564 10.2337/db23-0365 PMC10588284

[40] Aitkenhead, M. et al. Identification of endothelial cell genes expressed in an in vitro model of angiogenesis: induction of ESM-1, βig-h3, and NrCAM. *Microvasc Res.* **63** (2), 159–171 (2002). 11866539 10.1006/mvre.2001.2380

[41] Wang, X., Xu, L., Yu, Y. & Fu, Y. LncRNA RP5-857K21.7 inhibits PDGF-BB-induced proliferation and migration of airway smooth muscle cells through the miR-508-3p/PI3K/AKT/mTOR axis. *Autoimmunity.* **55** (1), 65–73 (2022). 34913773 10.1080/08916934.2021.1998895

[42] Zhang, J. & Ney, P. A. Role of BNIP3 and NIX in cell death, autophagy, and mitophagy. *Cell. Death Differ.* **16** (7), 939–946 (2009). 19229244 10.1038/cdd.2009.16 PMC2768230

[43] Ney, P. A. Mitochondrial autophagy: origins, significance, and role of BNIP3 and NIX. *Biochim. Biophys. Acta BBA - Mol. Cell. Res.* **1853** (10), 2775–2783 (2015). 10.1016/j.bbamcr.2015.02.022 25753537

[44] Marinković, M. & Novak, I. A brief overview of BNIP3L/NIX receptor-mediated mitophagy. *FEBS Open. Bio. ***11 **(12), 3230–3236 (2021). 10.1002/2211-5463.13307 PMC8634856 34597467

[45] Barycki, J. J. et al. Biochemical characterization and crystal structure determination of human heart short chain l -3-Hydroxyacyl-CoA dehydrogenase provide insights into catalytic mechanism. *Biochemistry.* **38** (18), 5786–5798 (1999). 10231530 10.1021/bi9829027

[46] Clayton, P. T. et al. Hyperinsulinism in short-chain L-3-hydroxyacyl-CoA dehydrogenase deficiency reveals the importance of β-oxidation in insulin secretion. *J. Clin. Invest.* **108** (3), 457–465 (2001). 11489939 10.1172/JCI11294 PMC209352

[47] Bennett, M. et al. Reye-like syndrome resulting from novel missense mutations in mitochondrial medium- and short-chain l-3-hydroxy-acyl-CoA dehydrogenase. *Mol. Genet. Metab.* **89** (1–2), 74–79 (2006). 16725361 10.1016/j.ymgme.2006.04.004

[48] Ott, G. et al. Functional characterization of protein variants encoded by nonsynonymous single nucleotide polymorphisms in *MARC1* and *MARC2* in healthy Caucasians. *Drug Metab. Dispos.* **42** (4), 718–725 (2014). 24423752 10.1124/dmd.113.055202

[49] Wu, X. & Levine, A. J. p53 and E2F-1 cooperate to mediate apoptosis. *Proc. Natl. Acad. Sci.* **91** (9), 3602–3606 (1994). 8170954 10.1073/pnas.91.9.3602 PMC43628

[50] Zhou, Z. et al. Granzyme A from cytotoxic lymphocytes cleaves GSDMB to trigger pyroptosis in target cells. *Science.* **368** (6494), 7548 (2020). 32299851 10.1126/science.aaz7548

[51] London, B. et al. Mutation in Glycerol-3-Phosphate dehydrogenase 1–Like gene (*GPD1-L*) decreases cardiac Na ^+^ Current and causes inherited arrhythmias. *Circulation.* **116** (20), 2260–2268 (2007). 17967977 10.1161/CIRCULATIONAHA.107.703330 PMC3150966

[52] Liu, M. et al. Cardiac Na ^+^ Current regulation by pyridine nucleotides. *Circ. Res.* **105** (8), 737–745 (2009). 19745168 10.1161/CIRCRESAHA.109.197277 PMC2773656

[53] Valdivia, C. R., Ueda, K., Ackerman, M. J. & Makielski, J. C. GPD1L links redox state to cardiac excitability by PKC-dependent phosphorylation of the sodium channel SCN5A. *Am. J. Physiol-Heart Circ. Physiol.* **297** (4), 1446–1452 (2009). 19666841 10.1152/ajpheart.00513.2009 PMC2770765

[54] Liu, M., Liu, H. & Dudley, S. C. Reactive oxygen species originating from mitochondria regulate the cardiac sodium channel. *Circ. Res.* **107** (8), 967–974 (2010). 20724705 10.1161/CIRCRESAHA.110.220673 PMC2955818

[55] Westaway, S. K. et al. Common Variants in *CASQ2*, *GPD1L*, and *NOS1AP* Are Significantly Associated With Risk of Sudden Death in Patients With Coronary Artery Disease. *Circ Cardiovasc Genet.* **4** (4), 397–402 (2011). 10.1161/CIRCGENETICS.111.959916 PMC3160237 21685173

[56] Mao, K. et al. Regulation of akt/pkb activity by P21-activated kinase in cardiomyocytes. *J. Mol. Cell. Cardiol.* **44** (2), 429–434 (2008). 18054038 10.1016/j.yjmcc.2007.10.016 PMC2278035

[57] Galambos, C. et al. Phenotype characterisation of *TBX4* mutation and deletion carriers with neonatal and paediatric pulmonary hypertension. *Eur. Respir J.* **54** (2), 1801965 (2019). 31151956 10.1183/13993003.01965-2018

[58] Thoré, P. et al. Phenotype and outcome of pulmonary arterial hypertension patients carrying a *TBX4* mutation. *Eur. Respir J.* **55** (5), 1902340 (2020). 32079640 10.1183/13993003.02340-2019

[59] Hernandez-Gonzalez, I. et al. West (eds) West J,. Clinical heterogeneity of pulmonary arterial hypertension associated with variants in TBX4. *PLOS ONE.* **15** (4), 0232216 (2020). 32348326 10.1371/journal.pone.0232216 PMC7190146

[60] Carter, D. B. et al. Purification, cloning, expression and biological characterization of an interleukin-l receptor antagonist protein. *Nature.* **344** (6267), 633–638 (1990). 10.1038/344633a0 2139180

[61] Steinberg, S. J., Wang, S. J., Kim, D. G., Mihalik, S. J. & Watkins, P. A. Human Very-Long-Chain Acyl-CoA synthetase: cloning, topography, and relevance to Branched-Chain fatty acid metabolism. *Biochem. Biophys. Res. Commun.* **257** (2), 615–621 (1999). 10198260 10.1006/bbrc.1999.0510

[62] Winkler, T. W. et al. A joint view on genetic variants for adiposity differentiates subtypes with distinct metabolic implications. *Nat. Commun.* **9** (1), 1946 (2018). 29769528 10.1038/s41467-018-04124-9 PMC5956079

[63] Papaioannou, V. E. The T-box gene family: emerging roles in development, stem cells and cancer. *Development.* **141** (20), 3819–3833 (2014). 25294936 10.1242/dev.104471 PMC4197708

[64] Pulit, S. L. et al. Meta-analysis of genome-wide association studies for body fat distribution in 694 649 individuals of European ancestry. *Hum. Mol. Genet.* **28** (1), 166–174 (2019). 30239722 10.1093/hmg/ddy327 PMC6298238

[65] Justice, A. E. et al. Protein-coding variants implicate novel genes related to lipid homeostasis contributing to body-fat distribution. *Nat. Genet.* **51** (3), 452–469 (2019). 30778226 10.1038/s41588-018-0334-2 PMC6560635

[66] Gesta, S. et al. Evidence for a role of developmental genes in the origin of obesity and body fat distribution. *Proc. Natl. Acad. Sci.* **103** (17), 6676–6681 (2006). 16617105 10.1073/pnas.0601752103 PMC1458940

[67] Piccolis, M. et al. Probing the global cellular responses to lipotoxicity caused by saturated fatty acids. *Mol. Cell.* **74** (1), 32–44 (2019). 30846318 10.1016/j.molcel.2019.01.036 PMC7696670

[68] Zhang, N. et al. Neutrophil degranulation and myocardial infarction. *Cell Commun Signal.* **20** (1), 50 (2022). 10.1186/s12964-022-00824-4 PMC8996539 35410418

[69] Lopaschuk, G. D., Ussher, J. R., Folmes, C. D. L., Jaswal, J. S. & Stanley, W. C. Myocardial fatty acid metabolism in health and disease. *Physiol. Rev.* **90** (1), 207–258 (2010). 20086077 10.1152/physrev.00015.2009

[70] Kolwicz, S. C., Purohit, S. & Tian, R. Cardiac metabolism and its interactions with contraction, growth, and survival of cardiomyocytes. *Circ. Res.* **113** (5), 603–616 (2013). 23948585 10.1161/CIRCRESAHA.113.302095 PMC3845521

[71] Shah, M. S. & Brownlee, M. Molecular and cellular mechanisms of cardiovascular disorders in diabetes. *Circ. Res.* **118** (11), 1808–1829 (2016). 27230643 10.1161/CIRCRESAHA.116.306923 PMC4888901

[72] Ritterhoff, J. & Tian, R. Metabolism in cardiomyopathy: every substrate matters. *Cardiovasc. Res.* **113** (4), 411–421 (2017). 28395011 10.1093/cvr/cvx017 PMC5852620

[73] Kraunsøe, R. et al. Mitochondrial respiration in subcutaneous and visceral adipose tissue from patients with morbid obesity. *J. Physiol.* **588** (12), 2023–2032 (2010). 20421291 10.1113/jphysiol.2009.184754 PMC2911209

[74] Mancuso, P. The role of adipokines in chronic inflammation. *ImmunoTargets Ther.* **5**, 47–56 (2016). 27529061 10.2147/ITT.S73223 PMC4970637

[75] Kwon, H. & Pessin, J. E. Adipokines mediate inflammation and insulin resistance. *Front. Endocrinol.* **4**, 71 (2013). 10.3389/fendo.2013.00071 PMC3679475 23781214

[76] Frenk, S. & Houseley, J. Gene expression hallmarks of cellular ageing. *Biogerontology.* **19** (6), 547–566 (2018). 29492790 10.1007/s10522-018-9750-z PMC6223719

[77] Ferrara, N. & Kerbel, R. S. Angiogenesis as a therapeutic target. *Nature.* **438** (7070), 967–974 (2005). 16355214 10.1038/nature04483

[78] Voiosu, A. M. et al. The diagnostic and prognostic value of serum endocan in patients with cirrhotic cardiomyopathy. *Rom J. Intern. Med.* **56** (3), 182–192 (2018). 29453929 10.2478/rjim-2018-0007

[79] Qiu, C. et al. Relationship of endothelial Cell–Specific molecule 1 level in stress hyperglycemia patients with acute ST-Segment elevation myocardial infarction: A pilot study. *Angiology.* **67** (9), 829–834 (2016). 26685180 10.1177/0003319715621996

[80] Qiu, C. R. et al. Analysis of serum endothelial Cell-Specific molecule 1 (Endocan) level in type 2 diabetes mellitus with acute ST-Segment elevation myocardial infarction and its correlation: A pilot study. *Angiology.* **68** (1), 74–78 (2017). 26927690 10.1177/0003319716634581

[81] Wei, P. et al. Association between Endothelial Cell-Specific Molecule 1 and Galectin-3 in Patients with ST-Segment Elevation Myocardial Infarction: A Pilot Study. *Oxid Med Cell Longev. ***2022**, 1723309 (2022). 10.1155/2022/1723309 PMC9646309 36388167

[82] Cui, P. et al. The novel axis of YAP1, transcription enhancer factor 3 and down syndrome candidate region 1 isoform 1L is a common signaling pathway downstream of several angiogenic factors. *Microvasc Res.* **129**, 103955 (2020). 31733305 10.1016/j.mvr.2019.103955

[83] Gollogly, L. K., Ryeom, S. W. & Yoon, S. S. Down syndrome candidate region 1-like 1 (DSCR1-L1) mimics the inhibitory effects of DSCR1 on calcineurin signaling in endothelial cells and inhibits angiogenesis. *J. Surg. Res.* **142** (1), 129–136 (2007). 17610901 10.1016/j.jss.2006.10.011 PMC1995402

[84] Hou, S. et al. A novel transcriptional complex on the VE-cadherin promoter regulated the downregulation of VE-cadherin in the down syndrome candidate region 1 isoform 1L-mediated angiogenesis. *Microvasc Res.* **138**, 104209 (2021). 34146582 10.1016/j.mvr.2021.104209 PMC9295908

[85] Miyazaki, T. et al. Calpastatin counteracts pathological angiogenesis by inhibiting suppressor of cytokine signaling 3 degradation in vascular endothelial cells. *Circ. Res.* **116** (7), 1170–1181 (2015). 25648699 10.1161/CIRCRESAHA.116.305363

[86] Wan, J., Che, Y., Kang, N. & Wu, W. SOCS3 blocks HIF-1α expression to inhibit proliferation and angiogenesis of human small cell lung cancer by downregulating activation of akt, but not STAT3. *Mol. Med. Rep.* **12** (1), 83–92 (2015). 25695729 10.3892/mmr.2015.3368 PMC4438922

[87] Zhang, X. Dysregulated Circulating SOCS3 and HP expression associated with stable CAD and acute coronary syndrome: an integrated study based on bioinformatics analysis and case control validation. *Anatol. J. Cardiol.* **24** (3), 160–174 (2020). 10.14744/AnatolJCardiol.2020.56346 PMC7585973 32870172

[88] Dokhanchi, M. et al. Colorectal cancer cell-derived extracellular vesicles transfer miR-221-3p to promote endothelial cell angiogenesis via targeting suppressor of cytokine signaling 3. *Life Sci.* **285**, 119937 (2021). 34508764 10.1016/j.lfs.2021.119937

[89] Zhou, S. et al. MiRNAS in cardiovascular diseases: potential biomarkers, therapeutic targets and challenges. *Acta Pharmacol. Sin*. **39** (7), 1073–1084 (2018). 29877320 10.1038/aps.2018.30 PMC6289363

[90] Li, P. et al. MicroRNA-663 prevents monocrotaline-induced pulmonary arterial hypertension by targeting TGF-β1/smad2/3 signaling. *J. Mol. Cell. Cardiol.* **161**, 9–22 (2021). 34339758 10.1016/j.yjmcc.2021.07.010

[91] Afonyushkin, T., Oskolkova, O. V. & Bochkov, V. N. Permissive role of miR-663 in induction of VEGF and activation of the ATF4 branch of unfolded protein response in endothelial cells by oxidized phospholipids. *Atherosclerosis.* **225** (1), 50–55 (2012). 22776647 10.1016/j.atherosclerosis.2012.06.016

[92] Li, P. et al. MicroRNA-663 regulates human vascular smooth muscle cell phenotypic switch and vascular neointimal formation. *Circ. Res.* **113** (10), 1117–1127 (2013). 24014830 10.1161/CIRCRESAHA.113.301306 PMC4537615

[93] Rashid, F., Awan, H., Shah, A., Chen, L. & Shan, G. Induction of miR-3648 upon ER stress and its regulatory role in cell proliferation. *Int. J. Mol. Sci.* **18** (7), 1375 (2017). 28661420 10.3390/ijms18071375 PMC5535868

[94] Margerie, D. et al. Hepatic transcriptomic signatures of Statin treatment are associated with impaired glucose homeostasis in severely obese patients. *BMC Med. Genomics*. **12** (1), 80 (2019). 31159817 10.1186/s12920-019-0536-1 PMC6545676

[95] Ziegler, A. K. & Scheele, C. Human adipose depots’ diverse functions and dysregulations during cardiometabolic disease. *Npj Metab. Health Dis.* **2 **(1), 34 (2024). 10.1038/s44324-024-00036-z PMC11606922 39619657

[96] Ou, M. Y., Zhang, H., Tan, P. C., Zhou, S. B. & Li, Q. F. Adipose tissue aging: mechanisms and therapeutic implications. *Cell. Death Dis. ***13 **(4), 300 (2022). 10.1038/s41419-022-04752-6 PMC8980023 35379822

