# Supplementary material for: Adipose tissue gene expression and longitudinal clinical phenotypes are early biomarkers of lipid-regulating drug usage
Source: Sci Rep. 2025 Aug 29;15:31861. doi: 10.1038/s41598-025-13693-x (PMC12397236; doi:10.1038/s41598-025-13693-x)
Supplement: Supplementary file 2 — Supplementary Material 2 [file 41598_2025_13693_MOESM2_ESM.docx]

**Supplementary figure 1.** The concordance of effect size estimates (LogFC) for limma-voom and DESeq2. A scatter plot comparison of the log_2_-fold change estimates for each gene from DESeq2 (x-axis) versus limma-voom (y-axis) differential expression analyses that compared controls and future lipid-regulating drug users. Points are coloured red if nominal (*p* < 0.05) in both methods. The black line displays the linear regression fit with an annotated R² value, indicating a strong agreement between these two methodological frameworks.

**Supplementary figure 2.** Exploring the variable importances from the highest performing machine learning models. A) Heatmap of feature importance for the top performing models generated by AutoML. Features on the y-axis, models on the x-axis, with colour intensity showing importance level. B) SHAP Summary plot of best performing XGBoost model in Combination 4 (ASCVD risk scores, 28 clinical phenotypes, and 1212 adipose gene expression at a nominal significance threshold). The colour gradient indicates normalised feature values per individual (dot) and SHAP value contributions to the overall model predictions.
